# Time-course analysis of cisplatin induced AKI in preclinical models: implications for testing different sources of MSCs

**DOI:** 10.1186/s12967-024-05439-6

**Published:** 2024-08-27

**Authors:** Abantika Ganguly, Shashank Chetty, Rosita Primavera, Steven Levitte, Shobha Regmi, Benjamin William Dulken, Scott M. Sutherland, Wendy Angeles, Jing Wang, Avnesh S. Thakor

**Affiliations:** 1 Interventional Radiology Innovation at Stanford (IRIS), Department of Radiology, School of Medicine, Stanford University, 3155 Porter Drive, Palo Alto, CA 94304 USA; 2 Department of Pathology, Stanford University, Palo Alto, CA USA; 3 Department of Pediatrics, Division of Nephrology, Stanford University, Palo Alto, CA USA

**Keywords:** Acute kidney injury, Cisplatin, Mesenchymal Stem Cells, Cell therapy, Transcriptomics, Temporal profile

## Abstract

**Background:**

Kidneys are at risk from drug-induced toxicity, with a significant proportion of acute kidney injury (AKI) linked to medications, particularly cisplatin. Existing cytoprotective drugs for cisplatin-AKI carry side effects, prompting a search for better biological therapies. Mesenchymal Stem Cells (MSCs) are under consideration given their regenerative properties, yet their clinical application has not achieved their full potential, mainly due to variability in the source of MSC tested. In addition, translating treatments from rodent models to humans remains challenging due to a lack of standardized dosing and understanding potential differential responses to cisplatin between animal strains.

**Method:**

In the current study, we performed a time-course analysis of the effect of cisplatin across different mouse strains and evaluated gender related differences to create a robust preclinical model that could then be used to explore the therapeutic efficacy of different sources of MSCs for their ability to reverse AKI.

**Result:**

Our data indicated that different mouse strains produce differential responses to the same cisplatin dosing regimen. Despite this, we did not observe any gender-related bias towards cisplatin nephrotoxicity. Furthermore, our time-course analysis identified that cisplatin-induced inflammation was driven by a strong CXCL1 response, which was used as a putative biomarker to evaluate the comparative therapeutic efficacy of different MSC sources in reversing AKI. Our data indicates that UC-MSCs have a stronger anti-inflammatory effect compared to BM-MSCs and AD-MSCs, which helped to ameliorate cisplatin-AKI.

**Conclusion:**

Overall, our data underscores the importance of using an optimized preclinical model of cisplatin-AKI to test different therapies. We identified CXCL1 as a potential biomarker of cisplatin-AKI and identified the superior efficacy of UC-MSCs in mitigating cisplatin-AKI.

**Graphical abstract:**

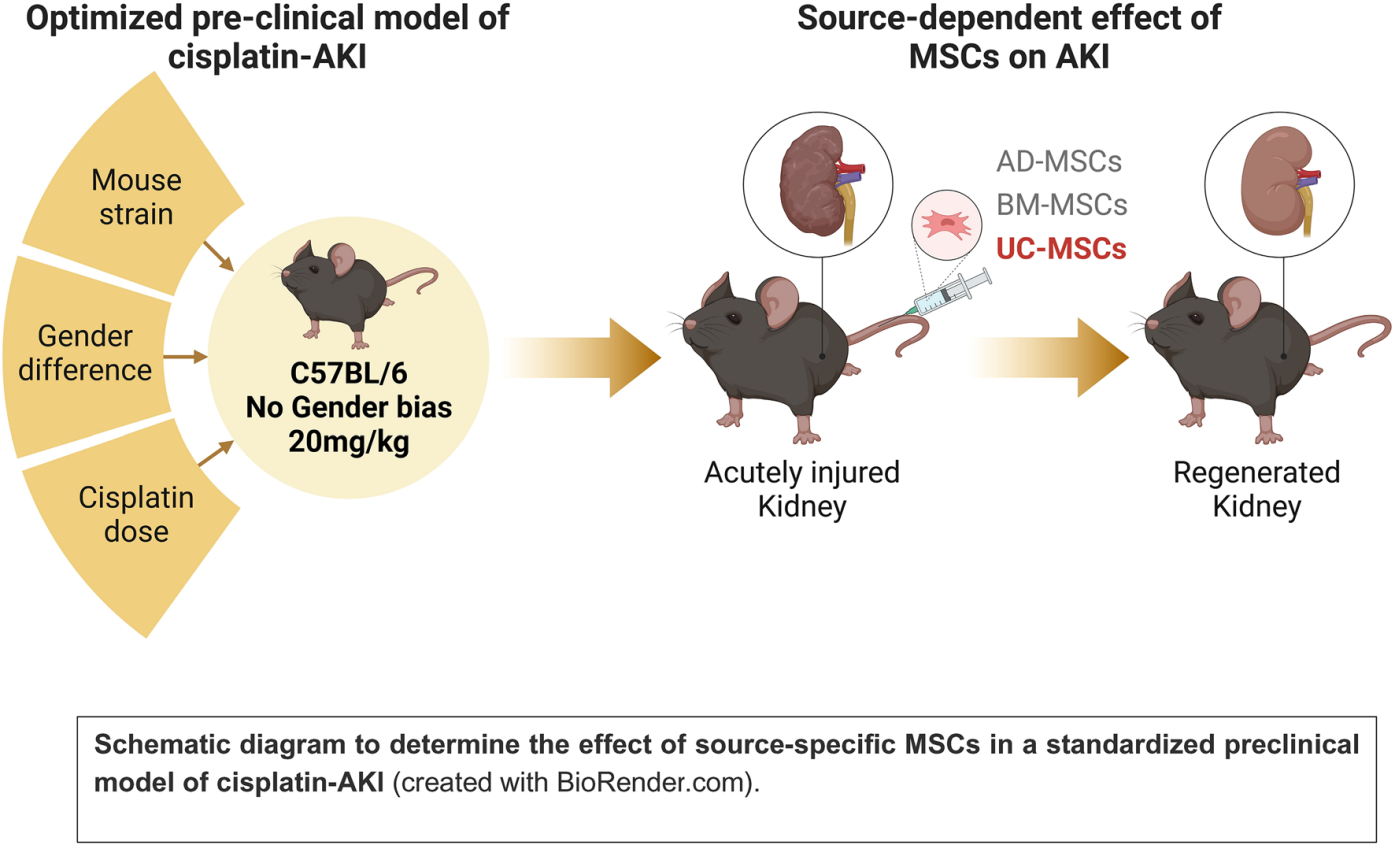

**Supplementary Information:**

The online version contains supplementary material available at 10.1186/s12967-024-05439-6.

## Background

The kidneys are frequently exposed to toxic drugs and metabolites and are therefore a common site for drug-related toxicity [1, 2]. Cisplatin remains the most effective and widely used treatment option for solid tumors [3], however, it is also responsible for 30–46% incidence of AKI in cancer patients [4]. The current standard of care for AKI is mostly supportive, with some patients requiring dialysis due to profound renal dysfunction [5], leading to high healthcare costs [6]. Thus, despite its broad anti-cancer potential, the clinical use of this chemotherapeutic agent remains limited [3]. Although several drugs have been approved clinically as cytoprotective agents for cisplatin-AKI, including OCT2 inhibitors [7] and antioxidant agents [8, 9], all of these drugs are associated with side effects including hypotension and hypocalcemia [10], which may restrict their universal usage for preventing cisplatin-AKI. These constraints have therefore spearheaded the need to look for alternative therapies for treating cisplatin related nephrotoxicity.

In the last decade, Mesenchymal Stem Cells (MSCs) have been proposed as a potentially useful therapeutic strategy in various diseases, including AKI [11]. Although MSC therapies have shown great promise in rescuing and regenerating the acutely injured kidney in pre-clinical models [12], their clinical translation has been sub-optimal, with some studies reporting lack of renal recovery, minimal impact on dialysis prevalence and comparable 30-day mortality between groups [13]. A key reason for this observed discrepancy between pre-clinical and clinical studies maybe attributed to the use of different sources of MSCs, which differ in their relative differentiation potential [14] and secretory profiles [15, 16], all of which contribute to the regenerative signature of MSCs. In a recent study, our group performed an integrated multi-omics based profiling of neonatal (umbilical cord-derived MSCs; UC-MSCs) and adult (bone marrow-derived MSCs; BM-MSCs and adipose tissue-derived MSCS; AD-MSCs) sources of MSCs [17] and identified source-specific differences in their anti-inflammatory, immunomodulatory, growth/proliferative and angiogenic properties, which collectively could have implications in the development of a targeted therapy for cisplatin-AKI.

Although, rodent models of cisplatin-induced toxicity remain the most reproducible with high clinical relevance, these preclinical models have failed to effectively translate novel treatment strategies for AKI into humans [18]. To improve the translatability of preclinical mice models of cisplatin-AKI, it is important to understand: (i) the effect of different dosing regimens, (ii) the role of gender-based differences, and (iii) the heterogeneity in the temporal profile of the functional and molecular responses between different strains of mice. Together, this will increase the likelihood of identifying and validating novel therapeutic targets for increased translatability into patients. Hence, in the present study, we looked at the dose-dependent responses to cisplatin in two different mouse strains, CD1 and C57BL/6 mice, and performed a transcriptome based time-course analysis of the molecular signature in male and female C57BL/6 mice during the progression of cisplatin-AKI, to generate a robust and reproducible preclinical model that could then be used to investigate the ability of different sources of MSCs to function as a rescue therapy to reverse AKI.

## Methods

### Cell culture

All MSCs were purchased from StemBioSys, USA (https://www.stembiosys.com/) and characterized based on the criteria by the International Society for Cell and Gene Therapy (ISCT) and expanded as previously described [17]. MSCs from passage 4–6 were used in this study. The HK-2 cell line was obtained from ATCC, USA (Catalogue: CRL-2190) and cultured in Keratinocyte Serum-free media (K-SFM) from Invitrogen (GIBCO) using additives 0.05 mg/mL Bovine Pituitary Extract (BPE) and 5 ng/ml human recombinant Epidermal Growth Factor (EGF).

### In vitro model of cellular injury in cisplatin-AKI

HK-2 cells were co-cultured with MSCs (in a trans well system) from different sources (n = 3 donors) in a ratio of 10:1 in serum-free media containing cisplatin at a concentration of 100 μM in saline (Sigma-Aldrich) for 24 h. For the determination of apoptosis, we used a colorimetric Cell Death Detection ELISA^PLUS^ (Roche, Switzerland) according to the manufacturer’s guidelines and absorbance was measured at 405 nm. Cellular ROS was measured using DCFDA (ThermoFisher USA) and fluorescence intensity was monitored at Ex/Em = 485/535 nm.

### Animal model

All animal experiments were performed according to the Institutional Animal Care and Use Committee (IACUC) at Stanford University. CD1 and C57BL/6 mice aged 6–8 weeks were acquired from Charles River Laboratories, USA. AKI was induced by a single intra-peritoneal injection of cisplatin (Sigma Aldrich, USA) in 0.5% saline at two different doses: 15 mg/kg and 20 mg/kg. AKI was defined by measurement of serum Creatinine > 0.3 mg/dL and 1.5-fold increase in BUN for two consecutive days. A single dose of MSCs (1 × 10^6^ cells/animal) was administered via tail vein injection at Day 3 following cisplatin injection (20 mg/kg). Animals were sacrificed on Day 5 or Day 7 based on experiments.

### Transcriptomics analysis

Transcriptomic analysis for all experimental groups was performed in C57BL/6 mice. For each time point both male (n = 4 biological replicates) and female mice (n = 4 biological replicates) were used. Paired end 150 bp sequencing was carried out using the Illumina HiSeq4000 platform. Alignment was performed against the Mouse Reference Genome GRCm38. The package DESeq2 was used to perform the differential gene expression (DEG) analysis and DEGs were considered significant using a threshold of Log [fold change] > 1.5 and p.adj (FDR) < 0.05. Hierarchical clustering was performed using uncentered correlation using Cluster 3.0 and temporal modules were identified by computing a similarity score between two joining elements/ dendrograms in the distance matrix with Correlation (R) > 0.1 considered as significant. Heatmaps were visualized using TreeView 3.0. Functional annotation of genes in each module was performed using The Database for Annotation, Visualization and Integrated Discovery (DAVID) [19, 20].

### Biochemical analysis

Serum blood urea nitrogen (BUN) and creatinine were assayed spectrophotometrically at the Stanford diagnostic facility. Total RNA was extracted using an RNAeasy kit (Qiagen, Germany) and RT-qPCR for gene expression analysis was performed using an iTaq Universal One-Step RT-qPCR Kit by SYBR GREEN method. Primers were procured from Integrated DNA Technologies (Table S2) and GAPDH was used as an internal control.

### Histopathology

Renal tissue was fixed in 10% neutral-buffered formalin and processed for light microscopy examination. Histopathological changes were analyzed by a pathologist following hematoxylin and eosin (H&E) staining. ROIs were defined as 1mm^2^ areas of renal parenchyma, and 10 ROIs were quantified per kidney.

### Statistical analysis

The data were analyzed using Prism 8 (Graphpad software). All in vivo experiments were performed with at least n = 4 biological replicates and in vitro studies performed using at least n = 6 biological replicates. To compare continuous variables among multiple independent groups, a non-parametric Kruskal–Wallis test was employed, to consider departures from normality in the distribution of the data. In cases where the Kruskal–Wallis test indicated a rejection of the null hypothesis of no differences among groups, post hoc pairwise comparisons were performed using the Two-stage linear step-up procedure of Benjamini, Krieger, and Yekutieli [23] to identify specific pairwise differences between the groups. To compare time-dependent variables among multiple groups, a mixed ANOVA was used followed by Sidak’s post hoc correction for evaluating pairwise comparisons between groups. A p-value (adjusted p-value for non-parametric and mixed ANOVA), P ≤ 0.05 was considered statistically significant. Efficacy for the different MSC-sources was calculated as percentage differences compared to untreated samples (Table S1).

## Results

### Development of a standardized preclinical model for cisplatin-AKI

Most preclinical studies evaluating cisplatin-AKI utilize a single sub-lethal dose of cisplatin which can induce severe AKI, characterized by increased BUN and serum creatinine levels. This model is useful for studying the acute inflammatory response of kidney injury without causing systemic toxicity and multi-organ failure, which clinically represents a different pathology. Based on published data for calculating mouse cisplatin dosing similar to human clinical dosing [21–23], doses in the range of 16–25 mg/kg were shown to induce moderate to severe AKI in > 50% of cases in mice following a single injection with no related mortality. Hence, for modeling cisplatin induced AKI we chose two different sub-lethal concentrations of cisplatin at 15 mg/kg and 20 mg/kg to test in our studies.

#### Effect of cisplatin dosing in different mouse strains

**Fig. 1:**
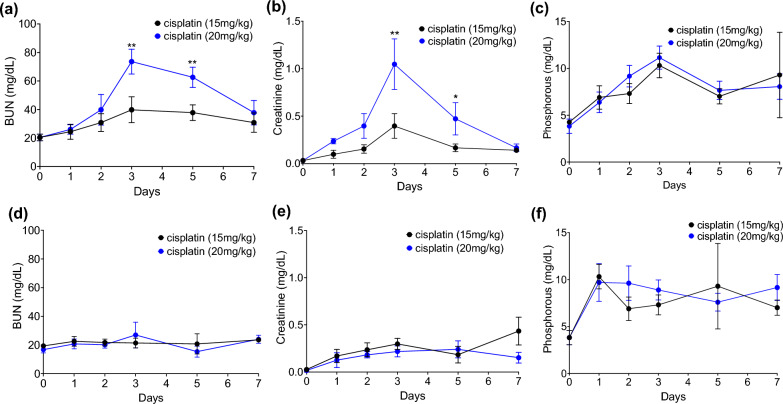
Time-course effect of cisplatin dosing in two different mouse strains. Panel showing changes in C57BL/6 renal functional parameters, serum (**a**) BUN, (**b**) Creatinine and (**c**) Phosphorous following intra-peritoneal (IP) injection of two different doses of cisplatin over 7 days. Panel showing changes in CD1 renal functional parameters, serum (**d**) BUN, (**e**) Creatinine and (**f**) Phosphorous following systemic injection of two different doses of cisplatin over 7 days. Statistical analysis: (**a**–**f**) Results expressed as mean ± SD (n = 5 biological replicates). Statistical significance was determined using a mixed ANOVA followed by Sidak’s post hoc correction for evaluating multiple pairwise comparison. *P ≤ 0.05; **P ≤ 0.01

AKI was induced in two different strains of mouse, CD1 and C57BL/6, using a single dose of cisplatin. CD1 and C57BL/6 mouse were injected with two different sub-lethal doses of cisplatin at 15 mg/kg and 20 mg/kg, and the effect on renal function was measured using serum BUN and creatinine over 7 days. In C57BL/6 mice, there was a gradual increase in serum BUN and creatinine levels with changes from baseline starting at Day 2 and sustained until Day 5, with a peak at Day 3 following both 15 mg/kg as well as 20 mg/kg doses. However, when comparing the maximal response at Day 3, the values for both serum BUN and creatinine were statistically more significant with 20 mg/kg dose compared to 15 mg/kg (Fig. 1a, b). Increase in blood phosphate levels, also followed a similar increase from baseline with a peak at Day 3; however, unlike BUN and creatinine levels, the hyperphosphatemia response did not show a dose-dependent effect with different cisplatin doses (Fig. 1c). In contrast, CD1 mice demonstrated only a small change from baseline for serum BUN, creatinine, and phosphate levels at Day 3 (Fig. 1d–f) following cisplatin injection, with no statistically significant difference observed between either cisplatin doses. Based on our observations, we identified that the C57BL/6 strain of mouse is more susceptible to cisplatin-induced nephrotoxicity, and 20 mg/kg of cisplatin was identified as the optimal dose to investigate the effect of gender on AKI severity and time-course progression of cisplatin-AKI.

#### Effect of gender on cisplatin-AKI induction

**Fig. 2:**
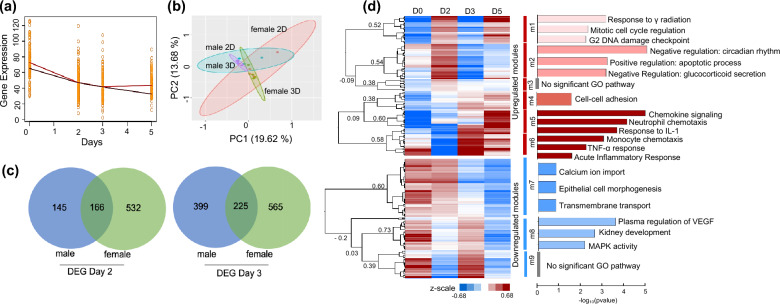
Time-course analysis of cisplatin-AKI on male and female mice. **a** Temporal profile of mean gene expression intensity in C57BL/6 male and female mice over a period of 5 Days following cisplatin dose with strong overlap between C57BL/6 male and female mice at Day 2 and Day 3 (Red: male; Brown: female). **b** PCA analysis of variance in gene expression data for C57BL/6 male and female mice at Day 2 and Day 3 following cisplatin injection (**c**) Venn diagram showing overlap of DEG [log(FC) > 1.5, FDR < 0.05] between C57BL/6 male and female mouse at Day 2 and Day 3. **d** Unsupervised hierarchical clustering of conserved DEGS from C57BL/6 male and female mouse at Day 2 and Day 3 identified n = 9 modules with a positive Pearson correlation (R) > 0.1. Heatmap visualization of temporal change in gene expression across each module. Modules were sorted into upregulated and downregulated based on their basal gene expression at Day 0. (D=Day). Top GO biological pathways for each module sorted by pvalue (p < 0.05). **a–d** All experiments were performed with male (n = 4 biological replicates) and female (n = 4 biological replicates)

To understand the progression of cisplatin-AKI at the cellular and molecular level, we performed a time-course analysis of kidney tissue from Day 0 to Day 5 in C57BL/6 mouse injected with 20 mg/kg cisplatin. To ensure an unbiased analysis, we also considered both male and female genders in our time-course analysis. Comparison of renal functional parameters alone, including serum BUN, creatinine and phosphate levels, between male and female mice over seven days following cisplatin injection did not indicate any statistically significant difference (Fig S1). In order to explore differences in response to cisplatin-AKI at the molecular level, we also performed a detailed transcriptomic profiling of changes in gene expression in male and female mice following 20 mg/kg cisplatin injection. A multi-variate PCA analysis following cisplatin injection indicated maximal variance in gene profile between healthy animals (D0) and those injected with cisplatin at Day 2 and Day 3, in both male and female mice (Fig S2a, b). Furthermore, the expression profile at Day 5 was similar to that of Day 0, but with some degree of overlap with Day 2 and Day 3, suggesting that complete molecular restoration is not achieved in AKI by Day 5 even when we observe a recovery of renal functions as measured by serum BUN and creatinine (Fig. 1). Temporal profile of mean gene expression intensity in male and female C57BL/6 mice showed a divergence between male and female healthy mice at Day 0 (Fig. 2a). Interestingly there was a convergence in the mean expression at Day 2 and Day 3, followed by a divergence at Day 5 (Fig. 2a). At the molecular level, PCA analysis was also used to confirm the low variance in kidney mRNA expression between male and female mice at Day 2 and Day 3 of cisplatin-AKI (Fig. 2b), suggesting that response to cisplatin-AKI may be similar, irrespective of gender. To further explore gender associated differences in cisplatin-AKI, a differential gene analysis was performed between males and females at Day 2 and Day 3, which identified a total of 239 upregulated and 392 downregulated DEGs between male and female mice respectively (Supplementary dataset S1). Gene Ontology (GO) analysis indicated differences in pathways related to steroid metabolic process, associated with sexual differentiation and reproduction (Fig S2c, d). We did not observe any gender-based differences in biological processes related to inflammatory response following cisplatin-AKI.

#### Time-course analysis of cisplatin-AKI progression

To identify the conserved genetic signature in cisplatin-AKI, significantly differentially expressed genes (DEGs) common between male and female mice at Day 2 (166 genes) and Day 3 (225 genes) were identified (Fig. 2c) and a time-course analysis with temporal module identification was undertaken using unsupervised hierarchical clustering (Fig. 2d, Supplementary dataset S2), which identified nine significant temporal modules with node value 0.3 ≤ R ≤ 1 (Fig. 2d, Supplementary dataset S2). Six of the modules (*m1–m6*) showed an upregulation of conserved DEGs compared to healthy animals at Day 0 and three modules (*m7–m9*) showed a downregulation of conserved DEGs compared to healthy animals at Day 0. GO analysis of genes in each module (Supplementary dataset S2) identified biological pathways related to DNA damage repair (*response to γ radiation*, *G2 DNA damage checkpoint*) and cell death (*apoptotic process, downregulation of glucocorticoid signaling*) as upregulated at Day 2, and at Day 5, while cytokine signaling (*Neutrophil and monocyte chemotaxis, TNFα and IL-1 response,*) was upregulated at Day 3 and remain upregulated into Day 5 following cisplatin administration. On the other hand, pathways related to epithelial cell damage (*epithelial cell morphogenesis*) and renal function (*calcium ion hemostasis, transmembrane transport*) were downregulated at Day 2, while pathways related to aberrant angiogenic signals associated with fibrosis (*VEGF signaling, MAPK kinase signaling*) were upregulated at Day 3. Taken together, the time-course analysis indicates that Day 3 following cisplatin administration to be the time point when the inflammatory milieu is most evident.

### Source-dependent effects of MSCs on alleviating cisplatin-AKI

**Fig. 3:**
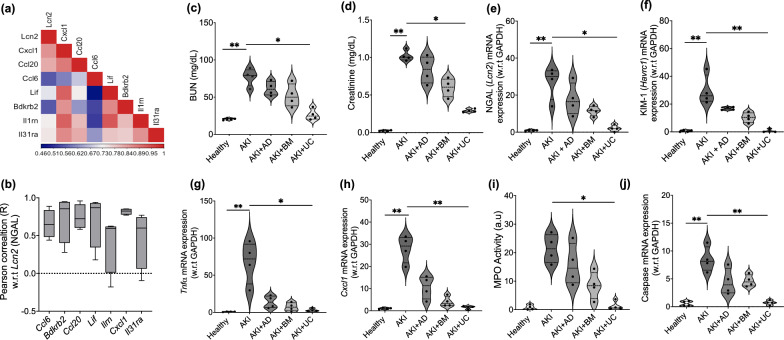
The effect of different sources of MSCs on renal function and inflammation following cisplatin-AKI. **a** Heatmap showing correlation of cytokines in the “Inflammation” module with the early kidney injury biomarker NGAL (*Lcn2*) at Day 3 which was the fixed timepoint of evaluation (**b**) Variation in Pearson correlation coefficient (R) for inflammatory cytokines w.r.t NGAL (*Lcn2*) over a period of 5 days. Changes in serum (**c**) BUN and (**d**) creatinine following cisplatin-AKI w/ and w/o treatment with different MSCs. Changes in mRNA expression level for early tubular injury biomarker (**e**) NGAL and (**f**) KIM-1 (*Havrc1*) following cisplatin-AKI w/ and w/o treatment with different MSCs. Changes in mRNA expression level of pro-inflammatory cytokines (**g**) *Tnfα* and (**h**) *Cxcl1* following cisplatin AKI w/ and w/o treatment with different MSCs. **i** Changes in Myleoperoxidase (MPO) enzyme activity following cisplatin AKI w/ and w/o treatment with different MSCs. **j** Changes in mRNA expression level of caspase in kidney tissue following cisplatin AKI w/ and w/o treatment with different MSCs. Statistical analysis: **c–j** Results are expressed as min to max data distribution with IQR range and median (n = 4 biological replicates). Statistical significance was determined using a non-parametric Kruskal–Wallis test, followed by 2-stage linear step-up procedure of Benjamini, Krieger, and Yekutieli for evaluating pairwise comparisons between the groups. *P ≤ 0.05; **P ≤ 0.01

To define the critical inflammatory signals that can be used as putative biomarker to follow the progression of cisplatin-AKI, we performed a Pearson correlation between the chemokines identified in our “Inflammation” module and the established biomarker for early kidney injury, NGAL (*Lcn2*), at Day 3. Interestingly, although all identified chemokines showed a positive correlation, the neutrophil chemoattractant *Cxcl1*, showed the highest correlation with NGAL (Fig. 3a). Furthermore, time-course based correlation of these chemokines with NGAL, indicated lowest variance for *Cxcl1* alone (Fig. 3b), which is in agreement with our GO biological pathways, indicating a critical role of neutrophils in cisplatin-AKI. Pro-inflammatory chemokines *Ccl20* and *Ccl6* with roles in monocyte chemotaxis were also found to share a strong positive correlation with NGAL, while anti-inflammatory cytokines *Il1rn, Il13ra* and *Lif* were found to have a weak correlation with NGAL during cisplatin-AKI progression. Thus, *Cxcl1* was identified as a putative inflammatory biomarker which could be used to investigate the efficacy of MSC therapies.

#### Effect of different sources of MSCs in reducing inflammation and improving renal function in cisplatin-AKI

Treatment with a single dose of different sources of MSCs at Day 3 improved renal function compared to untreated animals (Fig. 3c, d). The effect was source-dependent and UC-MSCs consistently reduced serum BUN and creatinine by 65–70% (Table S1), while AD-MSCs did not show any statistical significance. A similar source-dependent reduction was also observed with the kidney injury biomarker NGAL (*Lcn2*) and KIM-1 (*Havcr1*) (Fig. 3e, f). However, only UC-MSCs were found to be statistically significant in consistently reducing expression of these early kidney injury biomarkers by > 90% (Table S1). Furthermore, only UC-MSCs showed a statistically significant reduction in *Tnfα* levels from the untreated group by ≈ 96% (Fig. 3g) (Table S1). Expression levels for *Cxcl1* (Fig. 3h) also showed significant reduction from untreated levels by ≈ 96% when treated with UC-MSCs only (Table S1). A similar trend was also observed following estimation of the Myeloperoxidase Enzyme Activity (MPO) for activated neutrophils (Fig. 3i) as well as Caspase activation associated with cellular apoptosis (Fig. 3j).

#### Effect of different sources of MSC in alleviating renal tubular damage in cisplatin-AKI

**Fig. 4:**
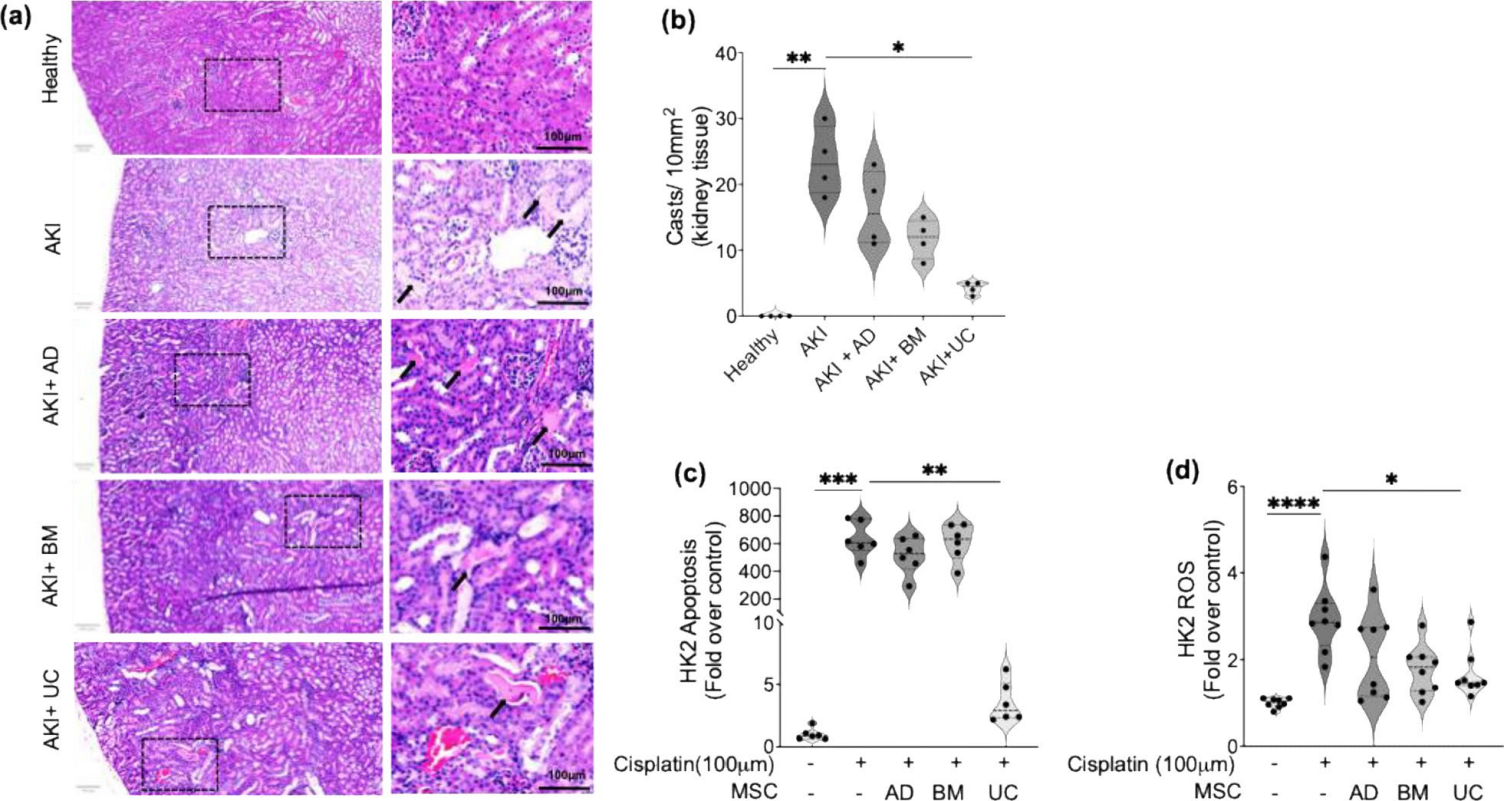
The effect of different sources of MSCs on renal tubular damage following cisplatin-AKI. **a** Representative H&E stained images of tubular cast formation following cisplatin-AKI and treatment with different sources of MSCs. **b** Quantification of tubular cast formation under the above-mentioned conditions. Changes in (**c**) apoptosis and (**d**) cellular ROS in HK-2 renal epithelial cells following cisplatin treatment w/ and w/o MSC therapy in our in vitro cellular model of AKI. Statistical analysis: **b** Results are expressed as min to max data distribution with IQR range and median (n = 4 biological replicates). Statistical significance was determined using a non-parametric Kruskal–Wallis test, followed by 2-stage linear step-up procedure of Benjamini, Krieger, and Yekutieli for evaluating pairwise comparisons between the groups. **c, d** Results are expressed as min to max data distribution with IQR range and median (atleast n = 6 biological replicates). Statistical significance was determined using a non-parametric Kruskal–Wallis test, followed by 2-stage linear step-up procedure of Benjamini, Krieger, and Yekutieli for evaluating pairwise comparisons between the groups. *P ≤ 0.05; **P ≤ 0.01; ***P ≤ 0.001 and ****P ≤ 0.0001

Renal tubular epithelial cells are one of the major sites for drug-related toxicity with the damage reflected as cast formation (cellular debris). Histopathological analysis indicated a reduction in renal tubular cast formation following treatment with MSCs (Fig. 4a), in a source-dependent manner, with UC-MSCs (75%) > BM-MSCs (52%) > AD-MSCs (23%) (Fig. 4b, Table S1). Furthermore, an in vitro cellular injury model involving cisplatin injury to human renal proximal tubular epithelial cells (HK-2 cells) indicated more than 600 × increase in cell apoptosis (Fig. 4c) and 3 × increase in cell ROS (Fig. 4d) following cisplatin-induced toxicity. While MSC therapies were able to reduce cellular apoptosis and ROS production to varying degrees in a source-dependent manner, UC-MSCs alone showed significant reduction in cell apoptosis in vitro compared to untreated conditions (Fig. 4c), while ROS production was reduced by both BM-MSCs and UC-MSCs compared to untreated conditions (Fig. 4d).

## Discussion

In the present study, we explored the dose-dependent responses to cisplatin in two different mouse strains, CD1 and C57BL/6 mice, and assessed gender and time-dependent heterogeneity in the molecular responses to cisplatin-AKI. Using this established preclinical model, we then investigated the ability of MSCs from different sources to rescue cisplatin-AKI.

To first optimize a relevant disease model, we used a single sub-lethal dose of cisplatin between 15 and 20 mg/kg, which is equivalent to a human cisplatin dosing regimen of 50–75 mg/m^2^, that has been clinically associated with a 50–75% incidence rate of mild-to-moderate AKI [21]. This allows us to closely mirror the human response to cisplatin nephrotoxicity, facilitating the development of a relevant and effective preclinical model for cisplatin-AKI. Next, we undertook comparisons between CD1 and C57BL/6 mice strains to better understand the effects of cisplatin induced nephrotoxicity in appropriate preclinical models. Despite previous work from our laboratory exploring the nephrotoxic effect of cisplatin on CD1 mice [24–26], which is also the preferred model for toxicology investigations [27, 28], we have consistently observed large variations in responses from this strain given inherent genetic and immunological diversity found in this strain [21, 29]. In contrast, C57BL/6 mice have been shown to have a more consistent immunological response [30], are sensitive to AKI from other causes [31] and are more genetically tractable thereby allowing for in depth pathway validation to be undertaken with this strain. Although other mouse strains have been studied in cisplatin-AKI, these were not evaluated in the present study given their limited applicability to AKI from other causes. These include the BALB/c and FVB/NJ mice strains, which are resistant to development of diabetic kidney disease [32] and its related complications, and 129/Sv mice strain, which is resistant to renal ischemia–reperfusion injury [33].

Currently, most preclinical studies exploring the effect of MSCs in cisplatin-AKI usually follow a single intravenous infusion of MSCs at 24 h following cisplatin injection [34, 35]. In contrast, based on our time-course analysis, we chose to treat animals with MSCs at Day 3 (i.e., 72 h) following cisplatin injection, given the traditional diagnostic biomarker for AKI, serum creatinine, was found to reach is maximal level at this time point. This time window enables the evaluation of rescue therapies to treat the maximal effect of cisplatin-AKI, while also helping to match the appropriate regenerative signature of source-specific MSCs to the pathophysiology driving the disease phenotype. Although 60–76% of cisplatin is eliminated by Day 2, a significant amount of the drug can be detected up to 21 days following a single dose [21]. In addition, cisplatin elimination has been shown to follow a biphasic curve [36], causing its concentration in the kidney to periodically peak. This can result in potential repeated damage to the kidney that, in turn, could then cause upregulation of DNA damage pathways that we observed at Day 2 and Day 5. Recent spatio-temporal transcriptomic analyses of kidney tissue following cisplatin induced nephrotoxicity has also identified differential DNA damage and regeneration patterns in different parts of the kidney at different time points [37]; however, in our study, we undertook bulk transcriptomic studies of the whole kidney tissue and thus could not accurately assess the temporal damage pattern to the different cell types, and hence this needs to be further evaluated in future studies.

The current clinical practice guidelines for AKI are based on recommendations provided by the Kidney Disease Improving Global Outcomes (KDIGO) [38], where severity of disease classification and AKI staging is based on the serum creatinine levels and estimated glomerular filtration rate (eGFR). Although of high clinical relevance, these indices lack sensitivity and specificity, thereby making early detection of AKI a challenge [39]. Among molecular biomarkers, which can used to predict and monitor subclinical stages of AKI, the early tubular injury biomarker—NGAL has gained prominence [40]. Nonetheless, work is still ongoing to develop novel and sensitive biomarkers for early AKI. Clinical and experimental studies have shown that AKI can generate a strong inflammatory response, however, there is less consensus in using inflammatory cytokines (TNFα, IL6 and IL18) as biomarkers for identifying AKI since such signals are also known to be involved in conditions of systemic stress. Notably, our time-course analysis suggests that when using a sub-lethal dose of cisplatin, which does not carry risk of systemic toxicity, CXCL1 may be a strong potential candidate biomarker to predict and monitor different stages of cisplatin-AKI, including the subclinical phase.

Overall, our data suggests that inflammation induced by cisplatin-AKI can be mitigated by all three sources of MSCs. However, among the different sources, only UC-MSCs were able to consistently decrease the expression of all pro-inflammatory cytokines, reduce tissue apoptosis and rescue kidney function by more than 90%. Previous work from our group on the differential regenerative potential of source-specific MSCs have shown that the secretome from UC-MSCs is enriched in the expression of anti-inflammatory molecules (i.e., IL4, IL13, IL12, IL35), compared to adult sources of MSCs (AD-MSCs and BM-MSCs) [17, 41]; these molecules can also effectively rescue cells from apoptosis and promote immune cell polarization to an anti-inflammatory phenotype. Incidentally, out of four clinicals trials listed on ClinicalTrials.gov. which use MSCs in AKI, including completed and unknown status, three of them (NCT00733876, NCT03015623 and NCT04445220) use BM-MSCs, while only one trial (NCT04194671) uses UC-MSCs, which likely contributes to the discrepancy noted in MSC efficacy observed in clinical trials. Taken together, our results underscore the importance of cell source selection as an important variable when considering how to translate MSCs into patients.

Overall, our study addresses the discrepancies in preclinical experimental models of cisplatin-AKI and identifies a source specific MSC therapy for potentially reversing cisplatin-AKI. Although promising, further validation is required with larger sample size to reduce the possibility of Type II error between various experimental groups [42]. In the current study, we have only focused on pro-inflammatory cytokines that were identified as significantly upregulated based on the temporal modules identified following transcriptomic profiling, which indicated the role of CXCL1 as a putative and novel biomarker for AKI. However, given the difference in timeline and dynamics of mRNA and protein regulation at the cellular level [43], a broader protein biomarker screen should be conducted to prevent a potential positive bias in biomarker identification. In addition to validating these findings, future studies should explore the differential effectiveness of MSCs from different sources in other models of kidney injury (i.e., ischemia–reperfusion) in order to prevent any model selection bias for the development of MSCs as a cell therapy for AKI. Although, our study aims to negate the effect of sampling bias by including both male and female sex in our study, future studies can also focus on exploring the effect of cisplatin-AKI and MSC therapy in animals of different ages, in order to prevent potential negative sampling bias. Future studies will also aim to evaluate potential cell-free options, in the form of extracellular vesicles derived from MSCs, that can overcome immunogenicity issues associated with cell therapies. Furthermore, although intravenous delivery is the most common technique used to administer MSCs into patients, this method results in a majority of cells getting trapped in the lung, requiring higher doses to ensure enough cells can then reach target organs. Future work will thus explore whether the regenerative potential of MSCs can be enhanced using precision delivery approaches (i.e., via locoregional intraarterial administration of MSCs directly into the kidney).

## Conclusion

In conclusion, our study highlights the importance for the development of an optimized preclinical model for understanding the pathophysiology of cisplatin-AKI. Our data shows the critical role which the chemokine CXCL1 plays in cisplatin-induced kidney inflammation, while also providing an opportunity for the evaluation of therapeutic interventions based on this, and other inflammatory biomarkers, in AKI. Notably, our comparative analysis of different sources of MSCs revealed distinct anti-inflammatory properties, with UC-MSCs exhibiting superior efficacy in mitigating cisplatin-AKI compared to BM-MSCs and AD-MSCs. Although preliminary, our findings are of interest as there are currently no approved therapies available to treat AKI of any etiology; the ability to mitigate AKI severity is relevant given that prolonged AKI can lead to future chronic kidney dysfunction.

### Supplementary Information

Supplementary Material 1.

Supplementary Material 2.

Supplementary Material 3.

## Data Availability

The authors declare that all data supporting the findings of this study are available within the article and its supplementary material files. The transcriptomics data has been deposited in GEO data set identifier GSE263678.

## Abbreviations

AKI: Acute kidney injury
MSC: Mesenchymal stem cells
DEG: Differentially expressed genes
GO: Gene ontology
NGAL: Neutrophil gelatinase associated lipocalin
KIM1: Kidney injury molecule-1
CCL20: Chemokine C–C motif ligand 20
CXCL1: Chemokine (C–X–C) ligand 1
OCT2: Organic cation transporter 2
ROS: Reactive oxygen species
