# Supplementary material for: Time-course analysis of cisplatin induced AKI in preclinical models: implications for testing different sources of MSCs
Source: J Transl Med. 2024 Aug 27;22:789. doi: 10.1186/s12967-024-05439-6 (PMC11348787; doi:10.1186/s12967-024-05439-6)
Supplement: Supplementary file 1 — Supplementary Material 1. [file 12967_2024_5439_MOESM1_ESM.pptx]

## Slide 1
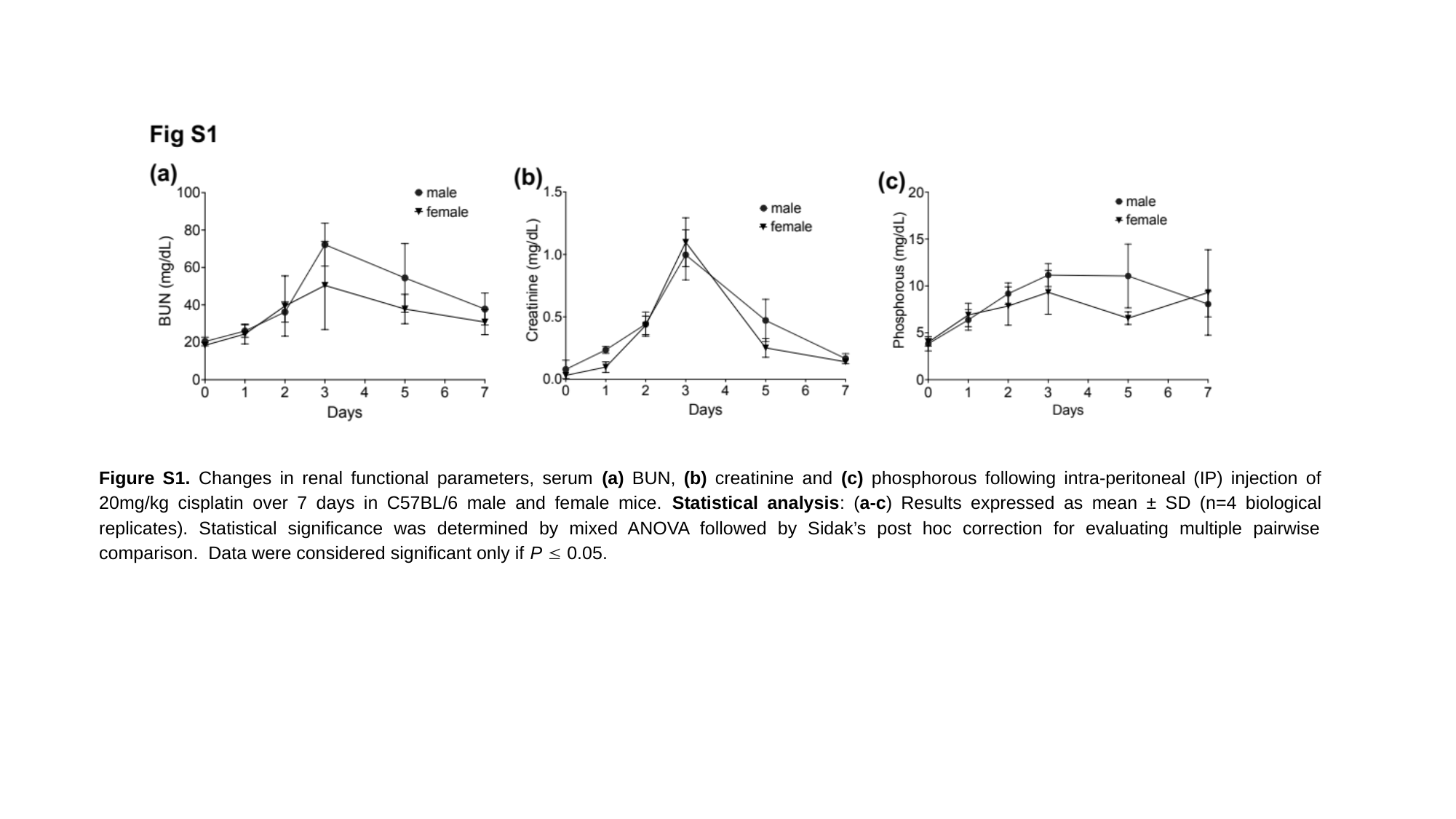

Figure S1. Changes in renal functional parameters, serum (a) BUN, (b) creatinine and (c) phosphorous following intra-peritoneal (IP) injection of 20mg/kg cisplatin over 7 days in C57BL/6 male and female mice. Statistical analysis: (a-c) Results expressed as mean ± SD (n=4 biological replicates). Statistical significance was determined by mixed ANOVA followed by Sidak’s post hoc correction for evaluating multiple pairwise comparison. Data were considered significant only if P ≤ 0.05.

## Slide 2
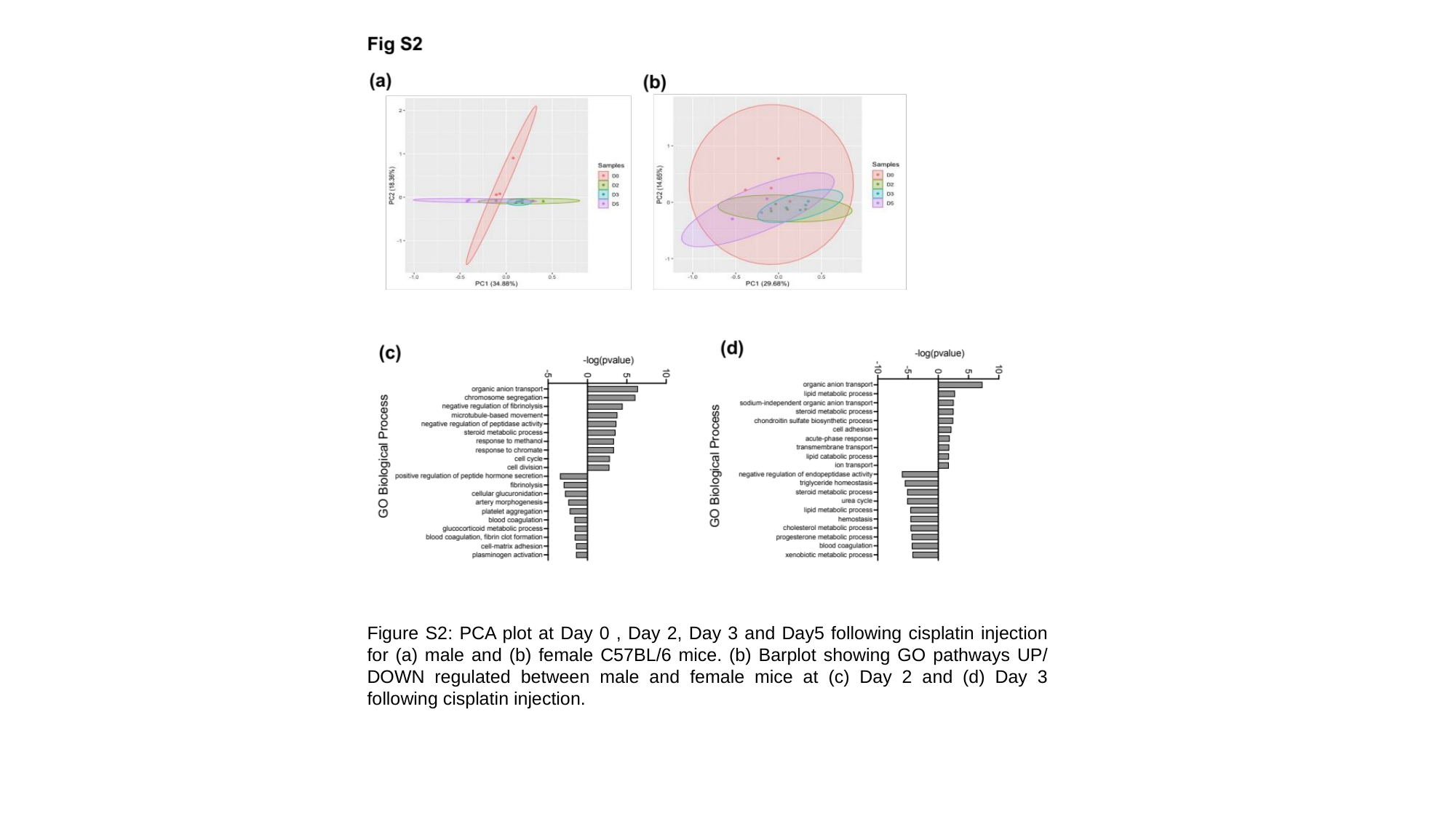

Figure S2: PCA plot at Day 0 , Day 2, Day 3 and Day5 following cisplatin injection for (a) male and (b) female C57BL/6 mice. (b) Barplot showing GO pathways UP/ DOWN regulated between male and female mice at (c) Day 2 and (d) Day 3 following cisplatin injection.

## Slide 3
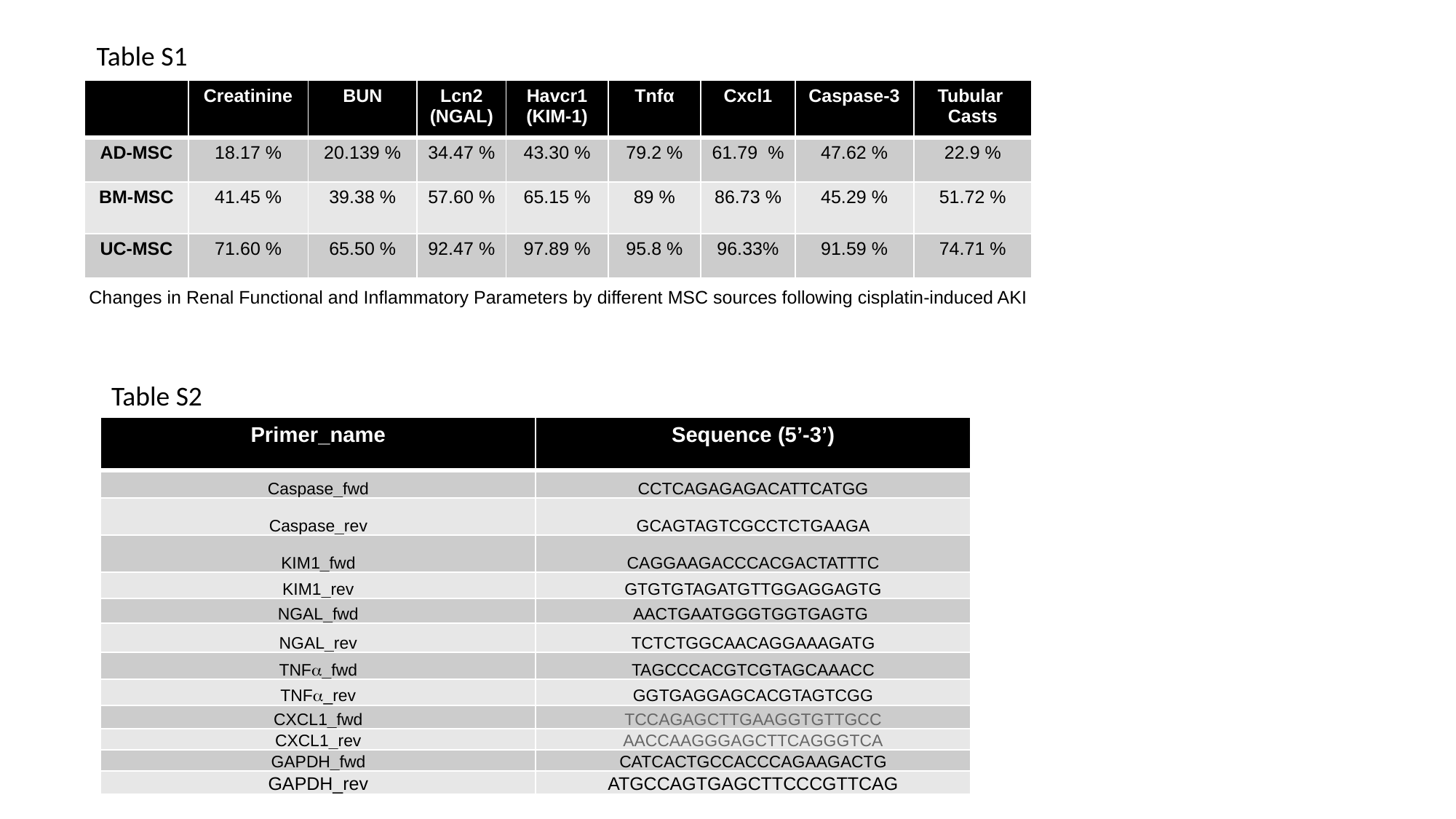

Table S1
| | Creatinine | BUN | Lcn2 (NGAL) | Havcr1 (KIM-1) | Tnfα | Cxcl1 | Caspase-3 | Tubular Casts |
| --- | --- | --- | --- | --- | --- | --- | --- | --- |
| AD-MSC | 18.17 % | 20.139 % | 34.47 % | 43.30 % | 79.2 % | 61.79 % | 47.62 % | 22.9 % |
| BM-MSC | 41.45 % | 39.38 % | 57.60 % | 65.15 % | 89 % | 86.73 % | 45.29 % | 51.72 % |
| UC-MSC | 71.60 % | 65.50 % | 92.47 % | 97.89 % | 95.8 % | 96.33% | 91.59 % | 74.71 % |
Changes in Renal Functional and Inflammatory Parameters by different MSC sources following cisplatin-induced AKI
Table S2
| Primer\_name | Sequence (5’-3’) |
| --- | --- |
| Caspase\_fwd | CCTCAGAGAGACATTCATGG |
| Caspase\_rev | GCAGTAGTCGCCTCTGAAGA |
| KIM1\_fwd | CAGGAAGACCCACGACTATTTC |
| KIM1\_rev | GTGTGTAGATGTTGGAGGAGTG |
| NGAL\_fwd | AACTGAATGGGTGGTGAGTG |
| NGAL\_rev | TCTCTGGCAACAGGAAAGATG |
| TNFα\_fwd | TAGCCCACGTCGTAGCAAACC |
| TNFα\_rev | GGTGAGGAGCACGTAGTCGG |
| CXCL1\_fwd | TCCAGAGCTTGAAGGTGTTGCC |
| CXCL1\_rev | AACCAAGGGAGCTTCAGGGTCA |
| GAPDH\_fwd | CATCACTGCCACCCAGAAGACTG |
| GAPDH\_rev | ATGCCAGTGAGCTTCCCGTTCAG |
